# Enhanced photocatalytic performance of WON@porous TiO_2_ nanofibers towards sunlight-assisted degradation of organic contaminants†

**DOI:** 10.1039/c8ra06477f

**Published:** 2018-09-21

**Authors:** Yahia H. Ahmad, Assem T. Mohamed, Mostafa H. Sliem, Aboubakr M. Abdullah, Siham Y. Al-Qaradawi

**Affiliations:** Department of Chemistry and Earth Sciences, College of Arts and Sciences, Qatar University Doha 2713 Qatar siham@qu.edu.qa; Center for Advanced Materials, Qatar University Doha 2713 Qatar

## Abstract

In the last few decades, TiO_2_ has been widely used in different types of photocatalytic applications. However, the relatively large optical band gap (∼3.2 eV), low charge carrier mobility and consequently its low quantum efficiency limit its photocatalytic activity. Herein, we construct a novel nanostructured heterojunction of WON/TiO_2_ nanofibers (NFs) by integration of TiO_2_ nanofibers synthesized by electrospinning of a polymer solution containing a titanium(iv) butoxide precursor with WON nanoparticles fabricated *via* annealing of a WO_3_ precursor in dry ammonia at 700 °C. The synthesized photocatalysts were characterized using different spectroscopic techniques. Their photocatalytic performance towards the degradation of methyl orange, methylene blue, and phenol as model contaminants was investigated and the charge transfer process was elucidated and compared to that of a TiO_2_/WO_3_ heterojunction.

## Abstract 2

Coupling of WON with TiO_2_ nanofibers creates a novel heterojunction with enhanced photocatalytic activity.

## Graphical abstract

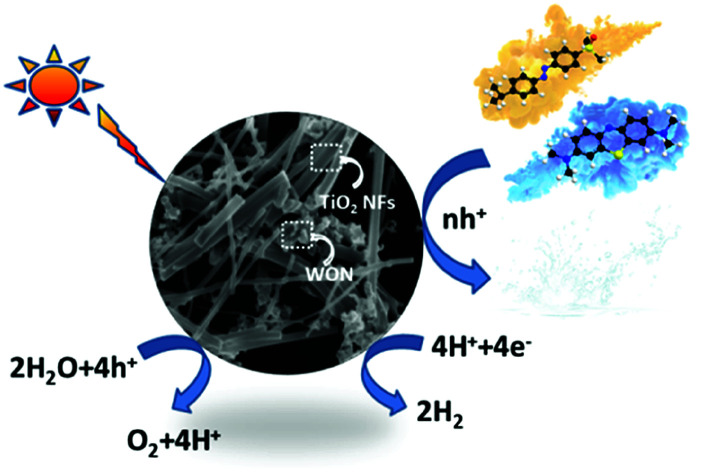

## Introduction

Environmental pollution caused by rapid worldwide industrial growth is considered as one of the most critical environmental issues especially those accompanied with bioaccumulation and toxicity. The lack of effective treatment at the contamination sources causes the release of hazardous materials into the water effluents. Some of these water contaminants such as heavy metal ions and organic dyes are extremely toxic and non-biodegradable. Different structures of organic dyes can exist in water which makes their removal a complicated issue. These dyes can undergo hydrolysis, oxidation or other chemical reaction which lead to formation of toxic by-products that severely affect human and aquatic life. Total removal of these dyes from water is a challenging issue due to their high solubility and high persistency. In this regard, different procedures have been devoted to decontaminate water from these hazardous materials. Among them, elector-coagulation, reverse osmosis, electro-oxidation, flocculation, adsorption and chemical oxidation. However, the aforementioned techniques have drawbacks of high cost in addition to the formation of deleterious by-products.

Recently, degradation of organic pollutants assisted only by solar energy as a clean renewable source has received considerable interest. In this respect, different types of photocatalysts have been devoted for this aim including plasmonic metals-based materials,^1–4^ metal–organic frameworks (MOFs),^5–10^ organic polymers based compounds,^11,12^ graphene and carbon nitride-based materials,^13–15^ metal complexes,^16,17^ hybrid compounds, and composites.^18–20^ Among them, metal oxides such as TiO_2_, WO_3_, CeO_2_, Cu_2_O and ZnO have been extensively used as photocatalysts for such application type.^21^ Owing to its chemical stability, rational resistance to photo-corrosion, high abundance, non-toxicity, and low cost, TiO_2_ is considered as one of the most widely used semiconductors in photocatalysis. In addition, TiO_2_ and TiO_2_ based catalysts can create hydroxyl radicals (·OH) that can efficiently degrade many pollutants. Several factors can influence the photocatalytic activity of TiO_2_, among them, the crystalline phase, morphology, surface area, exposed crystal facets, defects density, type of defects, and the degree of crystallinity.^22–24^ Nevertheless, the wide optical band gap (∼3.2 eV) which limits its absorption to UV region (∼4% sunlight), low electron mobility, short hole diffusion length, relatively fast recombination rate of photo-generated electron–hole pairs and consequently the low quantum efficiency lessen its photocatalytic performance.^25,26^ Different strategies have been devoted to decrease the band gap of TiO_2_ and enhance its photocatalytic performance, for instance, doping with metal^27–29^ and nonmetal,^30–32^ surface sensitization, coupling with other semiconductors, creation of lattice defects and fabrication of different morphologies, such as nanospheres,^33^ nanowires,^34^ nanofibers,^35^ nanotubes,^36,37^ nanorods,^38^ nanobelts,^39^ mesoporous TiO_2_,^40^ and 2D TiO_2_.^41^ Coupling of TiO_2_ with a semiconductor with more negative conduction band can greatly enhance photoactivity by improving the electron–hole separation, increasing the life time of charge carriers by decreasing the rate of charge recombination, and consequently facilitating the interfacial charge transfer between the semiconductor and the adsorbed molecules. Many semiconductors have been coupled with TiO_2_ to enhance its photocatalytic performance, among them, oxides such as ZnO,^42^ GeO_2_,^43^ Fe_2_O_3_,^44^ sulfides such as FeS_2_,^45^ MoS_2_,^46^ Cu_2_ZnSnS_4_,^47^ and wide variety of other semiconductors as BiVO_4_,^48^ C_3_N_4_,^49^ and CdSe.^50^ WO_3_ is a semiconductor with moderate band gap (∼2.8 eV), it is also chemically stable in acidic and nearly acidic media. It exhibits high rate of recombination of photo-generated charge carriers, the factor which greatly limits its performance in photocatalytic applications.^51,52^ Doping of WO_3_ with metal or non-metal can modify the band gap energy and hence widen its restricted absorption range from UV to visible region and enhance its photocatalytic activity as compared to WO_3_.^53^ Substitution of O with the less electronegative N to form oxynitride, WO_*X*_N_*Y*_ shifts the valence band to more negative potential due to contribution of N 2p in the valence band and consequently decreases the optical band gap.^54^ Different synthesis procedures have been devoted for the synthesis of tungsten oxynitride (WO_*X*_N_*Y*_) such as DC magnetron sputtering,^55^ metal–organic chemical vapor deposition (MOCVD)^56^ and the more common method, annealing under ammonia atmosphere.^57,58^ The performance of WO_*X*_N_*Y*_ in energy conversion and storage has been reported, however its application in photocatalysis was not reported before. Triggered by this, herein we reported facile synthesis of a novel TiO_2_/WON heterojunction by hybridization of nanoporous TiO_2_ nanofibers synthesized from electrospinning of polyvinylpyrrolidone solution of titanium(iv) butoxide with tungsten oxynitride (WO_*X*_N_*Y*_) synthesized by high temperature ammonolysis of WO_3_ precursor. The synthesized photocatalyst was characterized using different techniques UV-vis, SEM-EDX, TEM, BET, XRD, XPS, and Raman spectroscopy. Moreover, its photocatalytic activity for degradation of methyl orange, methylene blue, and phenol as model contaminants was also examined. Finally, a mechanism that accounts for the charge transfer and photocatalytic activity of WON/TiO_2_ was proposed compared to that of WO_3_/TiO_2_ heterojunction.

## Experimental

### Materials synthesis

#### Synthesis of TiO_2_ nanofibers (TiO_2_ NFs)

TiO_2_ precursor solution was prepared by dissolving tetrabutylorthotitanate (TBOT, 97%, Sigma-Aldrich) in a mixture containing 5 mL isopropanol and 3 mL acetic acid. Polymer solution was prepared by dissolving 1 g of polyvinyl pyrrolidone in 10 mL dimethyl formamide with continuous stirring till complete dissolution. TiO_2_ precursor solution was added dropwise to the polymer solution with vigorous stirring until a clear homogeneous solution is formed. The mixture was loaded into a plastic syringe with a capillary tip of diameter 0.1 mm. The solution was connected to a high voltage power supply. The solution was fed at a rate of 0.5 mL h^−1^ at a voltage of 15 kV was applied between the capillary tip and a collector aluminium foil held at a distance of 15 cm. The electrospun nanofibers were collected, dried at 80 °C for overnight and finally calcined in air at 450 °C for 3 h at a heating rate of 2 °C min^−1^.

#### Synthesis of WON

200 mg of tungsten(vi) oxide powder (WO_3_, Sigma-Aldrich, <100 nm) were loaded into a quartz boat and annealed in a tube furnace under a flow of dry ammonia at 700 °C for 2 h and a heating rate of 5 °C min^−1^ then cooled naturally to room temperature. The ammonia flow was kept at 200 mL min^−1^ throughout the whole experiment. Finally, the formed oxynitride was passivated in Ar gas containing 0.1% O_2_ for 1 hour at a flow rate of 500 mL min^−1^ before exposure to air.

#### Synthesis of TiO_2_/WON nanofibers (TiO_2_ NFs)

10 mg of as-synthesized WON was dispersed by sonication for 2 h in absolute ethanol. After dispersion TiO_2_ NFs were added to the solution which is re-sonicated for 30 min. The formed product was collected by 3 centrifugation/washing cycles and the dried in vacuum oven at 60 °C for 6 h. Three different compositions were synthesized 2.5% WON, 5% WON, and 10% WON, abbreviated as WON-2.5/TiO_2_, WON-5/TiO_2_, and WON-10/TiO_2_, respectively.

### Materials characterization

The morphology and composition of the as-synthesized materials was examined *via* field emission scanning electron microscope (FESEM, Philips XL-30, FEI Co., USA) equipped with an energy dispersive X-ray spectrometer (EDX). X-ray diffraction pattern, XRD was recorded using X'Pert-Pro MPD diffractometer (PANalytical Co., Netherlands) *via* using of Cu Kα X-ray source (*λ* = 1.54059 Å) as the X-ray source at the 2*θ* range 10–80. Raman spectra were investigated using a DXR 2 Raman Microscope (Thermo Fisher Scientific, USA) using a 780 nm laser source for excitation. The chemical composition and the valence states of elements were examined *via* XPS spectrophotometer Kratos Axis Ultra XPS equipped with a monochromatic Al Kα radiation source (1486.6 eV) under UHV environment (*ca.* 5 × 10^−9^ torr). All binding energies were calibrated to the C 1s peak at 284.8 eV. The BET surface area was obtained using N_2_ adsorption isotherms and samples were degassed for 24 h at 100 °C under vacuum before carrying out the measurements.

### Electrochemical impedance measurements

All electrochemical measurements were performed at room temperature into. A Pt wire, saturated calomel electrode (SCE) and the photocatalyst deposited on FTO glass were used as auxiliary, reference, and working electrodes, respectively. Electrochemical impedance spectroscopy (EIS) measurements were performed conventional three electrode cell using a Gamry electrochemical analyser (reference 3000, Gamry Co., USA) using AC voltage pulse with 5 mV amplitude in the frequency domain from 0.01 Hz to 100 kHz. Electrochemical impedance spectroscopy (EIS) measurements were carried out in each electrolyte solution at frequencies ranging from 0.1 Hz to 100 kHz with an AC voltage pulse of 10 mV amplitude.

### Photocatalytic activity measurements

Photocatalytic performance of different used photocatalysts was examined by degradation of methyl orange, MO and methylene blue, MB. A 100 W Xe arc lamp (13 013, Abet Technologies Inc., Milford, USA) was used as the light source. The light intensity was 100 mW cm^−2^. 50 mg of the photocatalyst was dispersed in an aqueous solution of MO (100 mL, 10 mg L^−1^), MB (100 mL, 10 mg L^−1^), and phenol (100 mL, 40 mg L^−1^) Prior to the light irradiation, the mixtures were magnetically stirred for 30 min in dark in order to reach the adsorption–desorption equilibrium between the photocatalyst and synthetic dye. At different time intervals, aliquots (4 mL) of the test solution were sampled and centrifuged to remove the photocatalyst, then concentration was monitored by measuring the absorbance at *λ*_max_ 463 nm, 664 nm, and 270 nm for MO, MB, and phenol, respectively *via* UV-vis spectrophotometer (Agilent 8453, China).

## Results and discussion

**Fig. 1:**
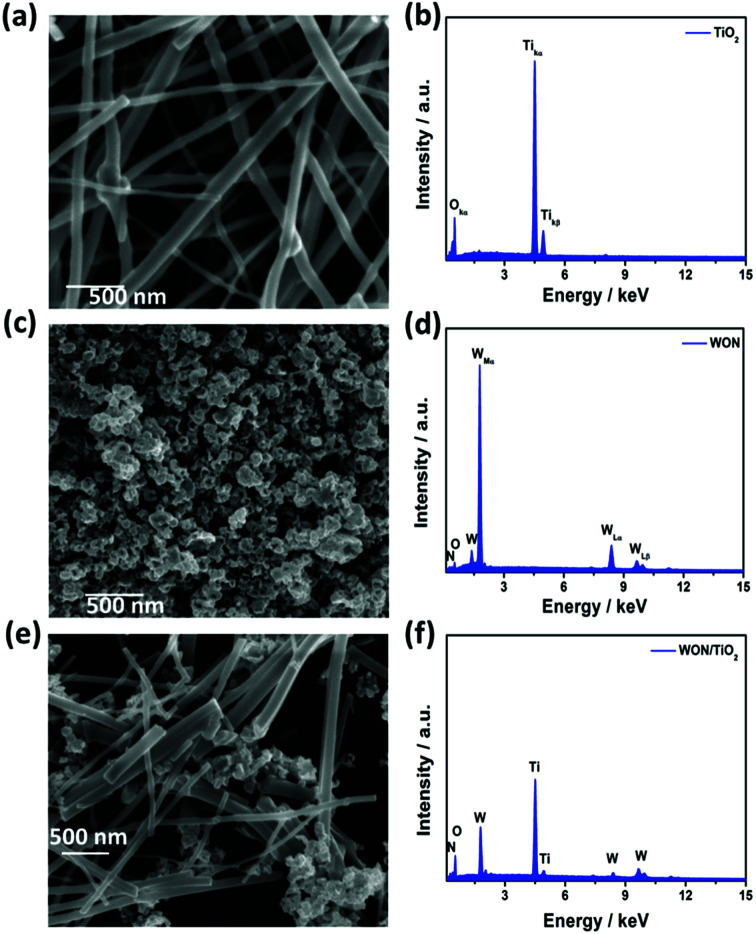
SEM Images of (a) TiO_2_ NFs, (c) WON, and (e) WON/TiO_2_ and EDX Analysis of (b) TiO_2_ NFs, (d) WON, and (f) WON/TiO_2_.

Fig. 1a FE-SEM image of the electrospun TiO_2_ NFs after annealing in air at 450 °C for 2 h. TiO_2_ NFs show high aspect ratio with uniform smooth surfaces with few beads. The length of fibers ranges from few micrometers to tens of micrometers, whereas their diameters show wide distribution ranging from 40 to 150 nm.

**Fig. 2:**
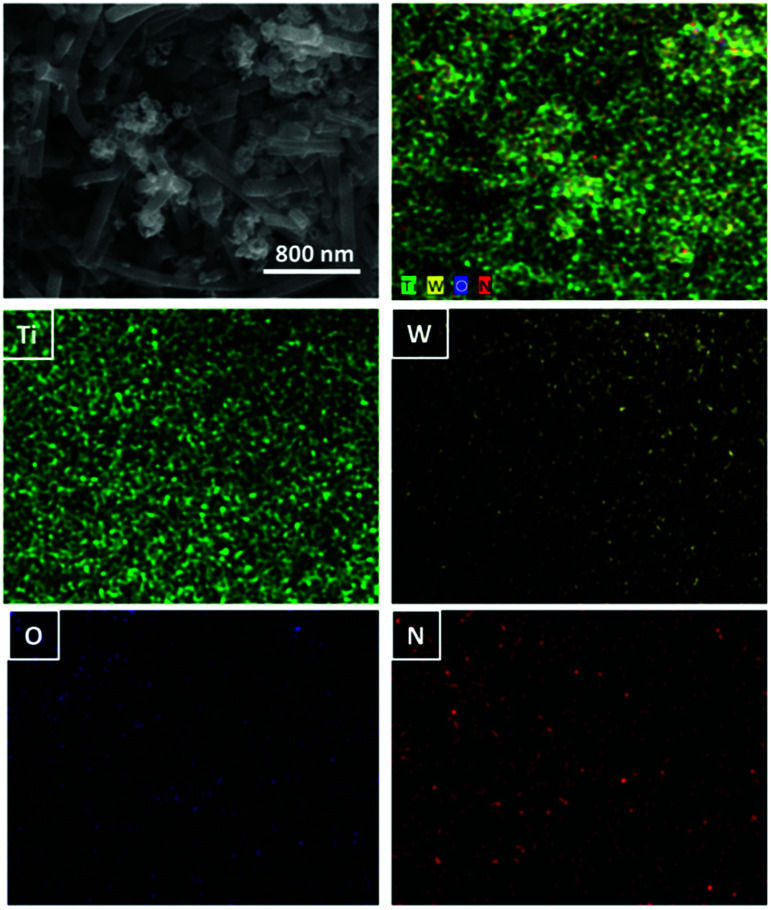
EDX mapping analysis of WON/TiO_2_.

Fig. 1c exhibits the SEM image of WON nanoparticles synthesized by ammonolysis of WO_3_ nanopowder. The particles exhibit distorted morphology with average particle size less than 100 nm. WON/TiO_2_ micrograph (Fig. 2e) manifests distribution of WON aggregates among TiO_2_ nanofibers with significant decrease in the length of TiO_2_ nanofibers due to ultrasonication effect.

EDX analysis of different materials was conducted to investigate the elemental composition of different photocatalysts. EDX spectrum of TiO_2_ nanofibers (Fig. 2b) revealed a composition of Ti : O 31.7 : 68.3, and that of WON expressed a composition of (W : O : N 42.8 : 29.7 : 27.5). WON/TiO_2_ expressed elemental composition of (Ti : W : O : N 30.8 : 2.6 : 64.3 : 2.3) with well distribution of different elements throughout the composite (Fig. 2).

**Fig. 3:**
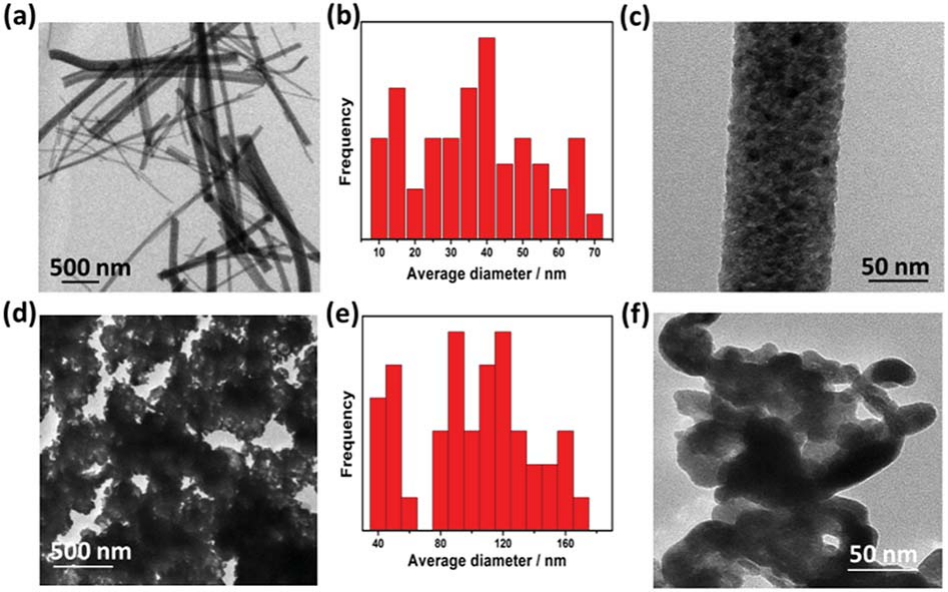
TEM low resolution images (a and d), particle size distribution (b and e), and high resolution images (c and f) of TiO_2_ NFs and WON, respectively.

**Fig. 4:**
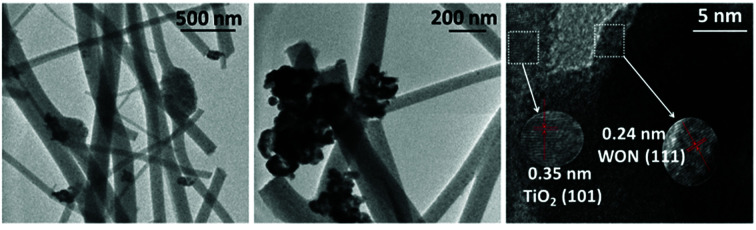
TEM and lattice resolved HR-TEM of WON/TiO_2_.

TEM image of TiO_2_ (Fig. 3a) indicates that annealed TiO_2_ showed a wide distribution of diameter ranging from 40 to 170 nm with average diameter of 104 nm (Fig. 3b). HR-TEM image (Fig. 3c) confirms the nanoscale porosity of annealed fibers. TEM image of WON particles showed nanosized particles with average diameter of 37 nm (Fig. 3e) interconnected together in large aggregates (Fig. 3d and f). Lattice resolved HR-TEM image of WON/TiO_2_ displayed well defined lattice fringes of WON and TiO_2_ which were assigned to (111) facet of WON with *d*-spacing of 2.3 Å and (101) of TiO_2_ with *d*-spacing of 3.5 Å (Fig. 4).

**Fig. 5:**
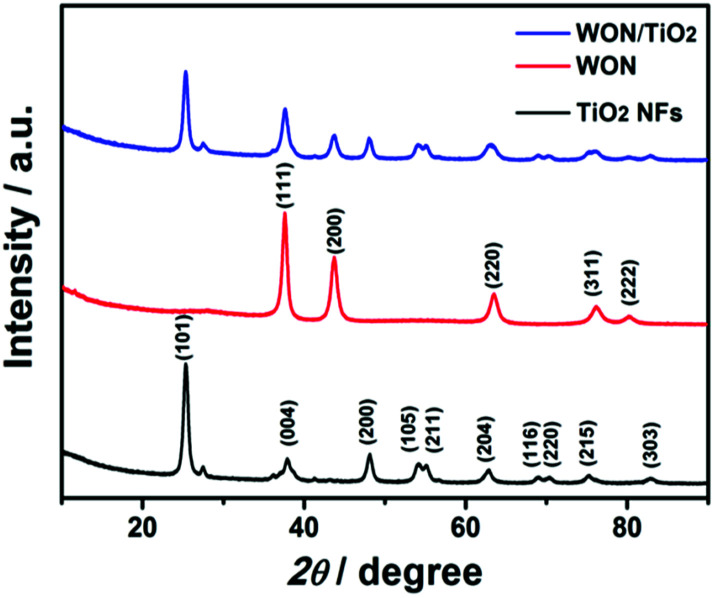
XRD spectra of TiO_2_ NFs, WON, and WON/TiO_2_.

The X-ray diffraction pattern of TiO_2_ NFs, WON, and WON/TiO_2_ composite depicts a well crystalline structures (Fig. 5). The diffraction pattern of TiO_2_ confirms the presence of anatase as a predominant phase (JCPDS no. 00-021-1271) with small diffraction peaks at 27.4° and 36.1° assigned to (110) and (101) planes of rutile phase, respectively. The diffraction pattern of WON matches that of face centered cubic W_0.62_(N,O) (JCPDS no. 025-1254). The XRD of WON/TiO_2_ matches the integrated pattern of TiO_2_ and WON with slight decrease in the crystallite size as revealed from increased values of FWHM of the diffraction peaks.

Raman spectroscopy is hired to investigate the crystallinity and the formation of chemical bonds.^59^ Fig. S1† presents the Raman spectra of TiO_2_ NFs, WON, and WON/TiO_2_ (all details are available in ESI† Section).

**Fig. 6:**
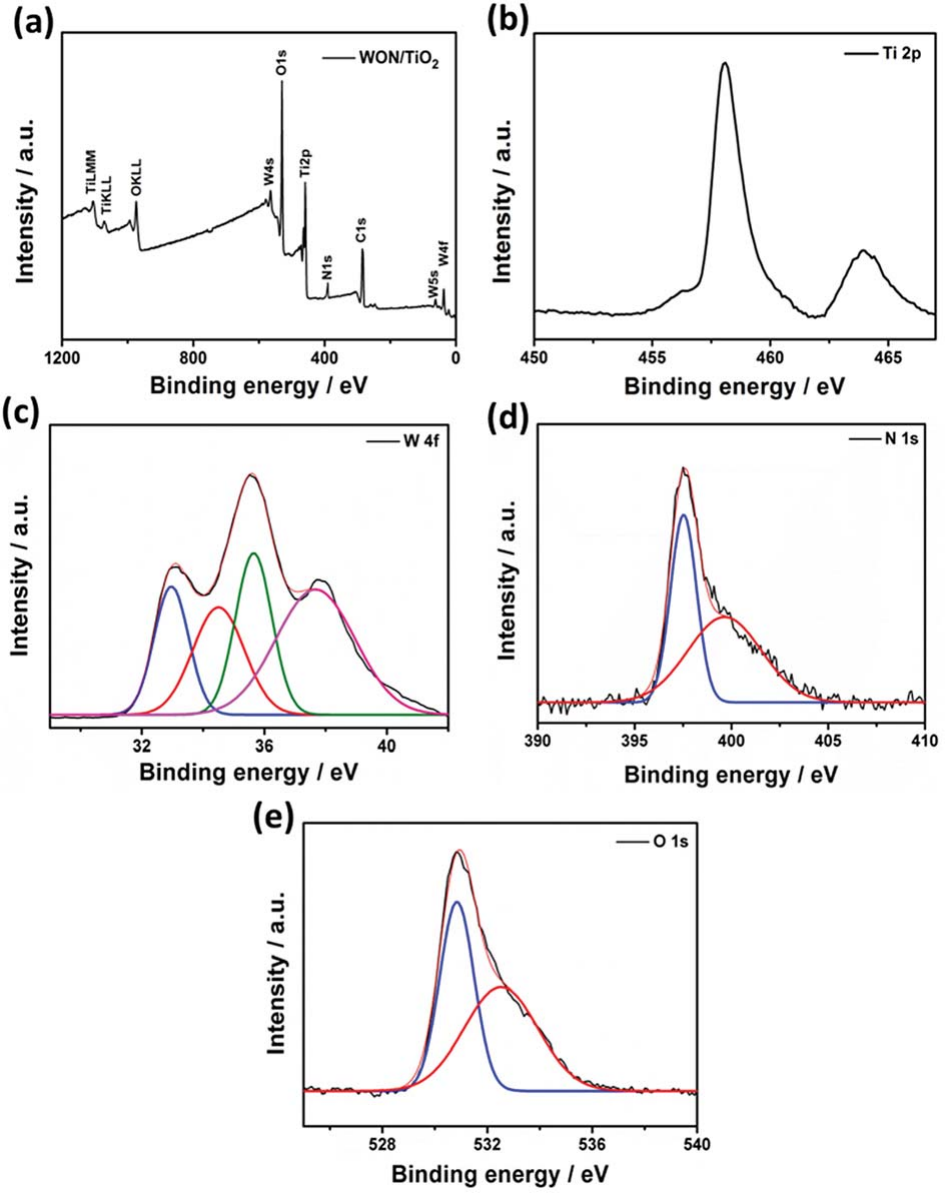
XPS spectrum of (a) WON/TiO_2_ and high resolution spectra of (b) Ti 2p, (c) W 4f, (d) N 1s, and (e) O 1s.

The surface composition, chemical bonding and oxidation states of WON/TiO_2_ heterojunction was investigated compared to TiO_2_ NFs and WON using XPS. Fig. 6a shows the survey spectrum of WON/TiO_2_ composite, it demonstrate the existence of Ti and O as main constituents and W and N as minor elements, in addition to presence of surface adsorbed carbon. C 1s peak at 284.6 eV was used for calibration. High resolution spectrum of Ti displayed a well resolved doublet at 458.3 and 464.0 eV corresponding to Ti 2p_3/2_ and Ti 2p_1/2_, respectively (Fig. 6b). They are assigned to Ti^4+^ valence state in TiO_2_.^60^ The high resolution XPS spectrum of tungsten W 4f (Fig. 6c) was deconvoluted into four peaks. Two at 37.6 and 35.6, corresponding to 4f_5/2_ and 4f_7/2_, respectively and assigned to W^6+^ oxidation state and the other two peaks at 34.6 and 33.0 eV attributed to lower oxidation state W^5+^ of oxynitride.^58^

Two different types of nitrogen can be detected from the XPS of N 1s (Fig. 6d). Intense peak at 397.5 eV corresponding to N bonded to W–O and a smaller peak at 400.0 eV assigned to surface adsorbed nitrogen and/or nitrogen trapped in the surface layers as nitrogen.^61^ Deconvolution of high resolution XPS spectrum of oxygen reveals two peaks at 530.8 and 532.5 eV corresponding to lattice oxygen O^2−^, and oxygen bonded to nitrogen (O–N), respectively (Fig. 6e).^62,63^ The binding energy of Ti 2p was shifted to higher binding energies in the composite WON/TiO_2_ relative to TiO_2_ NFs which confirms the interaction between mixed photocatalysts in the composite (Fig. S3†). In addition the binding energies of W 4f_7/2_ and 4f_5/2_ were shifted to lower values in WON/TiO_2_ confirming the interaction between TiO_2_ and WON (Fig. S4†).^64^

**Fig. 7:**
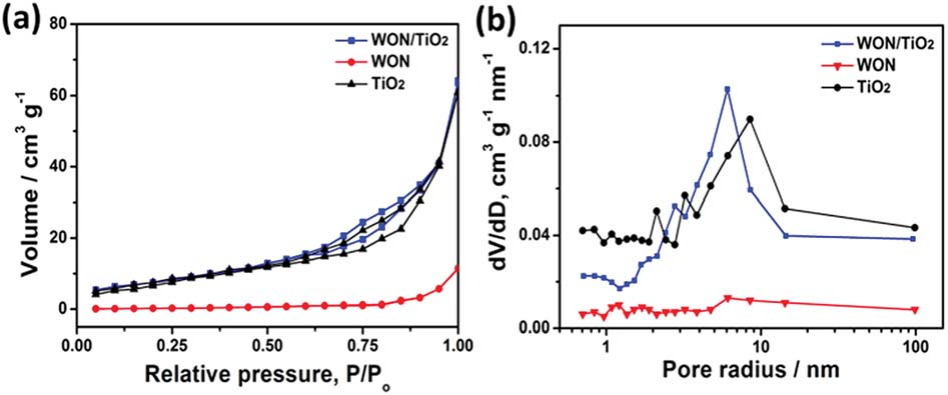
(a) Adsorption/desorption isotherm curves, and (b) BJH pore size curves of TiO_2_ NFs, WON, and WON/TiO_2_.

Fig. 7a represents N_2_ adsorption/desorption isotherm curves of TiO_2_ NFs, WON, and WON/TiO_2_. The calculated specific surface area of WON/TiO_2_, TiO_2_ NFs, and WON were 58.22, 52.82, and 8.54 m^2^ g^−1^, respectively, whereas the calculated pore size of WON/TiO_2_ and TiO_2_ NFs was 6.11 and 8.53 nm, respectively (Fig. 7b). The increased specific surface area of WON/TiO_2_ can create more active sites which ease the access of reactant molecules and hence enhance the photocatalytic activity.

**Fig. 8:**
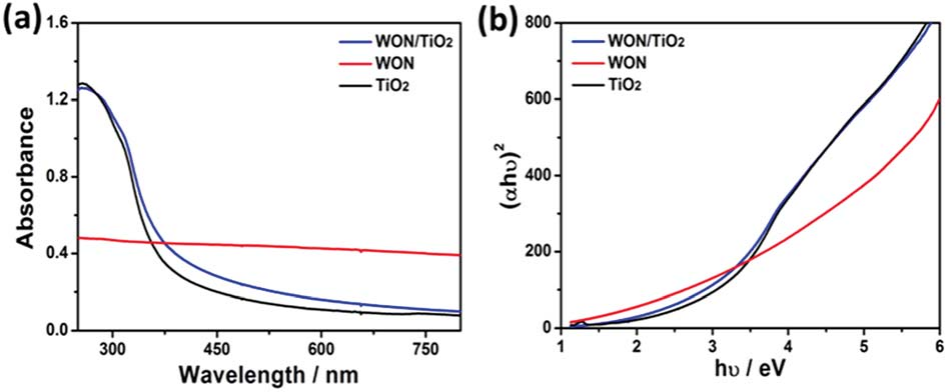
UV-vis absorption spectra (a) of TiO_2_ NFs, WON, and WON/TiO_2_ and Tauc plots (b) of TiO_2_ NFs, WON, and WON/TiO_2_.

The optical properties of the synthesized photocatalysts were investigated using UV-vis absorption spectroscopy (Fig. 8). TiO_2_ NFs showed absorption edge at 384 nm whereas WON-5/TiO_2_ exhibited slight shift in the absorption edge at 398 nm. The absorption spectrum of WON show no absorption edge. It shows light absorption over the whole visible range and this is indicated by its black color. The optical band gap of semiconductors can be calculated using Tauc plots from UV-vis absorption spectrum.

The band gap, *E*_g_ can be calculated by:*αhν* = *A*(*hν* − *E*_g_)^1/2^where *A* is a constant, *h* is Planck's constant, *ν* is the light frequency, and *α* is the absorption coefficient. The band gap energy, *E*_g_ can be calculated by extrapolating the tangent line of the plot between (*αhν*)^2^*vs.* photon energy, *hν*. TiO_2_ NFs expressed a band gap of 3.22 eV, while the band gap of the WON-5/TiO_2_ exhibited a slight reduction and found to be 3.09 eV. This slight decrease in the optical band gap energy can improve the photocatalytic performance of WON/TiO_2_ compared to bare TiO_2_.

**Fig. 9:**
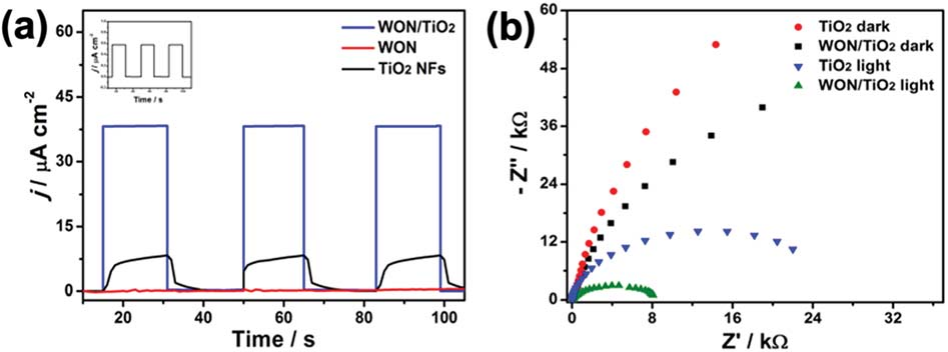
(a) Transient it responses of different photoelectrodes without bias in 0.1 M Na_2_SO_4_ solution, WON in the inset and (b) Nyquist impedance plots of TiO_2_ and WON/TiO_2_ electrodes at open-circuit potential in 0.1 M Na_2_SO_4_ solution.

Transient photocurrents *vs.* time responses of different photoelectrodes in 0.1 M Na_2_SO_4_ solution under simulated sunlight illumination are displayed in Fig. 9a. WON/TiO_2_ photoelectrode expressed better photoswitching with faster response time and enhanced photostability than TiO_2_ photoelectrode. The coupled photoelectrode also displayed enhanced photocurrent density which is 4.6 and 635 times more greater than TiO_2_ and WON photoelectrodes, respectively. The extremely low photocurrent density expressed by WON despite its low band gap confirms its poor photo-induced charge separation and consequently faint photocatalytic activity.

Electrochemical impedance spectroscopy, EIS was hired to investigate the charge transfer process at WON/TiO_2_ and TiO_2_ photo-electrodes. Fig. 9b displays Nyquist plots of WON/TiO_2_ and TiO_2_ in 0.1 M Na_2_SO_4_ solution at open circuit potential under dark and simulated solar light illumination. The recorded Nyquist plot of WON/TiO_2_ exhibited a lower semicircle diameter than TiO_2_ demonstrating decreased charge transfer resistance and lower conductivity. This proves that the heterojunction facilitates charge separation and transportation relative to pure TiO_2_.^65^

**Fig. 10:**
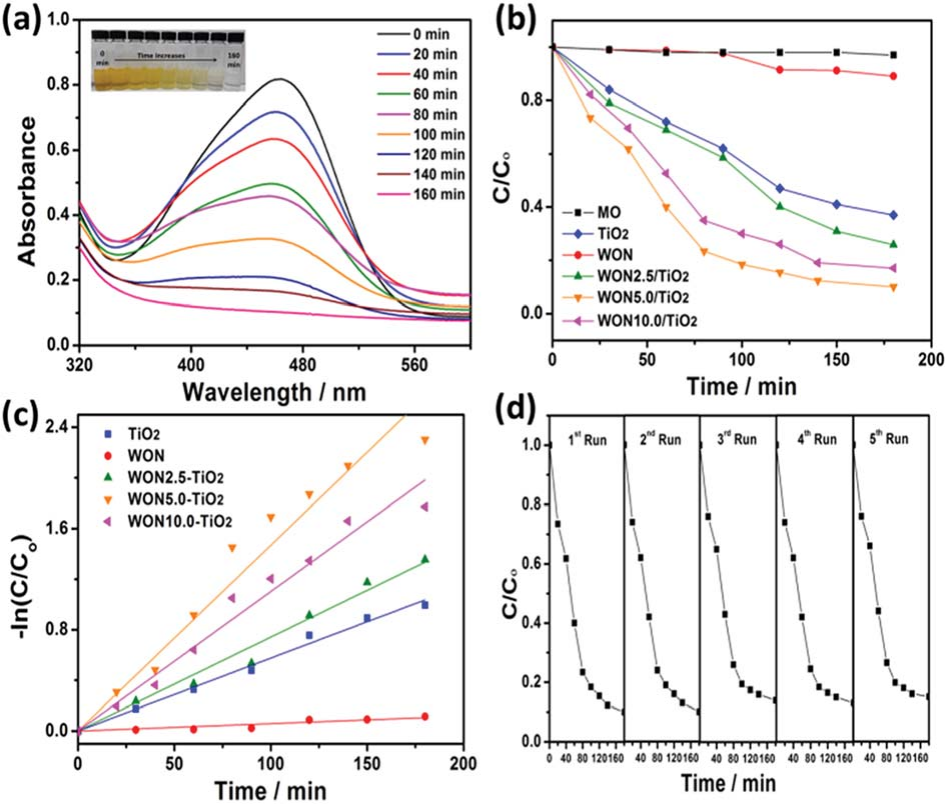
(a) UV-vis absorption spectra of MO degradation over WON-5/TiO_2_, (b) photocatalytic degradation efficiencies of MO over different photocatalysts under sunlight irradiation, (c) linear kinetic fit for MO degradation with different photocatalysts, and (d) reusability of WON-5/TiO_2_ towards degradation of MO.

The photocatalytic performance of the synthesized photocatalysts was evaluated by studying degradation of MO and MB as model contaminants. The absorption spectra of MO recorded in the presence of WON-5/TiO_2_ is given in Fig. 10a. The spectra were recorded from 400–700 nm. From the measured spectra, the maximum absorbance at 464 nm decreases gradually with the irradiation time indicating the degradation of MO.

The degradation efficiency can be evaluated as *C*/*C*_o_, where *C* and *C*_o_ are defined as the remnant and initial concentration of the dye, respectively. Fig. 10b represents the photo-degradation rate of MO under simulated sunlight in the absence and presence of TiO_2_ NFs, WON, and their mixtures in different ratios particularly, 2.5%, 5%, and 10% WON. No detectable photo-degradation was observed for MO over a time interval of 180 min in absence of photocatalyst, which confirms the stability of MO under UV-vis light irradiation and that self-photolysis is negligible during the course of degradation process. Only 10% of MO was degraded in WON after 180 min confirming the weak photocatalytic performance of WON despite its relatively small band gap energy which can be attributed to fast annihilation of the photo-generated hole–electron pairs. Notwithstanding the relatively larger band gap of TiO_2_ compared to WON, but its photocatalytic activity is higher due to relatively larger life time of charge carriers than WON. Obviously, WON-5/TiO_2_ exhibited the optimum composition with the best photocatalytic performance among all studied photocatalysts. The degradation ratio of MO over WON-5/TiO_2_ reached 90% after 180 min under simulated sunlight irradiation, while it needed 180 min for WON-10/TiO_2_ and WON-2.5/TiO_2_ to reach about 83% and 74%, respectively. TiO_2_ and WON expressed degradation rates of 63%, and 11%, respectively after the same time interval (Fig. 10b).

The photodegradation of the studied dyes follows the first order kinetics model: ln(*C*/*C*_o_) = *kt* where *k* is the degradation rate constant. The estimated rate constants for the used photocatalysts are 5.92 × 10^−4^, 5.75 × 10^−3^, 7.4 × 10^−3^, 1.47 × 10^−2^, and 1.1 × 10^−2^ min^−1^ for WON, TiO_2_, WON-2.5/TiO_2_, WON-5/TiO_2_, and WON-10/TiO_2_, respectively (Fig. 10c). The rate constant of WON-5/TiO_2_ is almost 25 times as greater as WON and 2.5 times that of TiO_2_. The photo-stability of a photocatalyst can be evaluated by measuring its recycle degradation performance for the contaminant. The first five degradation cycles of WON-5/TiO_2_ show no significant change in the degradation efficiency of WON-5/TiO_2_ towards photo-degradation of MO (Fig. 10d).

**Fig. 11:**
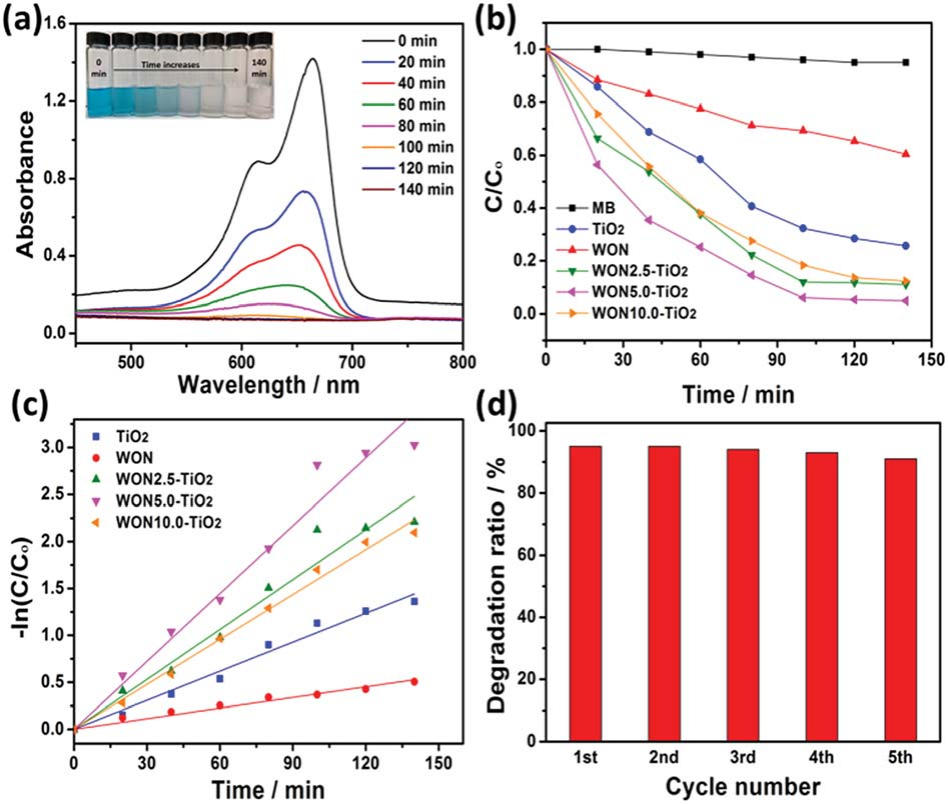
(a) UV-vis spectra of MB degradation over WON-5/TiO_2_, (b) photocatalytic degradation efficiencies of MB over different photocatalysts under sunlight irradiation, (c) linear kinetic fit for MB degradation with different photocatalysts, and (d) successive photodegradation cycles of MB over WON-5/TiO_2_.

Typical absorption spectrum of MB in presence of WON-5/TiO_2_ is given in Fig. 11a. The maximum absorbance at 664 nm decreases gradually with irradiation time indicates the decrease of MB concentration as a result of degradation. The rate of decay of MB over different expressed as *C*/*C*_o_*vs. t* is given in Fig. 11b. No significant degradation was observed under light irradiation in absence of photocatalyst during the course of experiment which confirms that MB is stable against self-photolysis at the selected range of wavelength. Obviously, WON-5/TiO_2_ expressed the optimum photocatalytic activity compared to other counterparts. For instance, after 100 min of light irradiation, WON-5/TiO_2_ manifested highest photocatalytic activity with 94% decay for MB, whereas WON-2.5/TiO_2_, WON-10/TiO_2_, TiO_2_, and WON exhibited 89, 88, 74, and 39% decay at the same time interval.

The plot of ln(*C*/*C*_o_) *vs.* time for different photocatalysts showed straight lines with a slope equals the rate constant of the photo-degradation reaction, *k* (Fig. 11c). The values of calculated rate constants for WON, TiO_2_, WON-2.5/TiO_2_, WON-5/TiO_2_, and WON-10/TiO_2_ are 0.0038, 0.0103, 0.0177, 0.02408, and 0.0159 min^−1^, respectively. Once more, WON-5/TiO_2_ show the highest rate constant which is almost 2.5 and 6.5 times as greater as that of TiO_2_ and WON, respectively.

Durability of the photocatalyst is a key factor that determines the possibility of its commercialization. Repeating of MB degradation experiments for five times showed no considerable decay in the degradation efficiency of WON-5/TiO_2_ which confirms its stability (Fig. 11d).

**Fig. 12:**
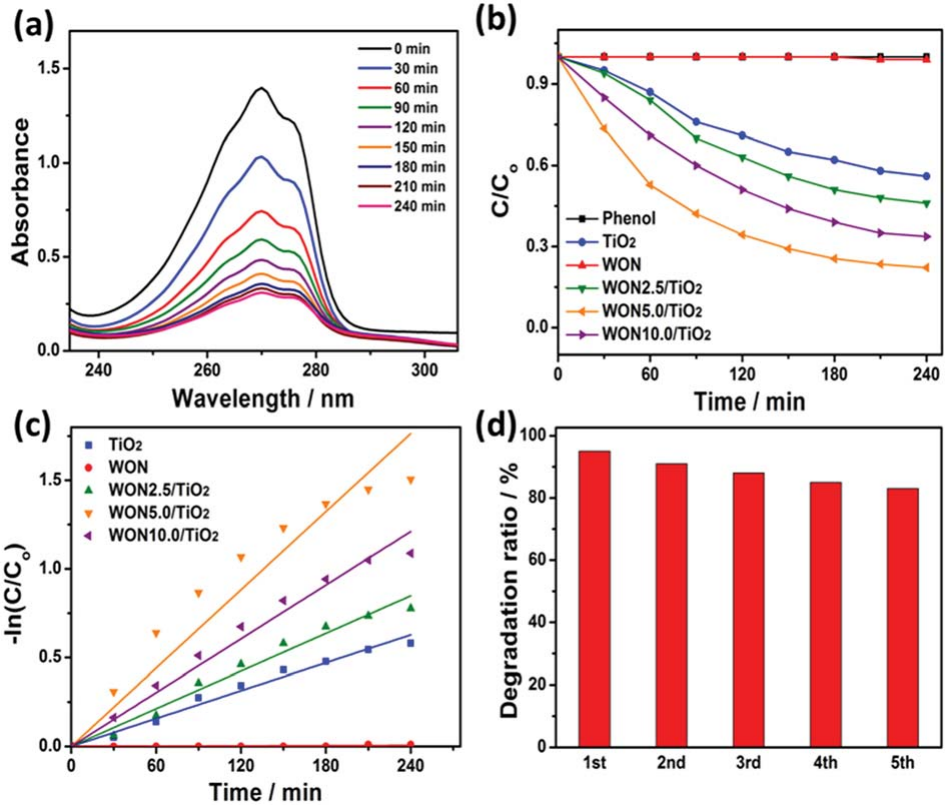
(a) UV-vis spectra of phenol degradation over WON-5/TiO_2_, (b) photocatalytic degradation efficiencies of phenol over different photocatalysts under sunlight irradiation, (c) linear kinetic fit for phenol degradation with different photocatalysts, and (d) successive photodegradation cycles of phenol over WON-5/TiO_2_.

The photocatalytic performance of WON/TiO_2_ was also examined in the photodegradation of phenol as a model hazardous contaminant. Typical absorption spectra were obtained for WON-5/TiO_2_ in phenol solution with maximum absorption at 270 nm of decreasing intensity with time (Fig. 12a).

Plotting of *C*/*C*_o_*vs.* time for different photocatalysts confirms the enhanced photocatalytic activity of WON-5/TiO_2_ over its counterparts (Fig. 12b). In addition, the estimated rate constant, *k* for different photocatalysts were 2.46 × 10^−5^, 2.61 × 10^−3^, 3.53 × 10^−3^, 7.35 × 10^−3^, and 5.05 × 10^−3^ min^−1^ for WON, TiO_2_, WON-2.5/TiO_2_, WON-5/TiO_2_, and WON-10/TiO_2_, respectively (Fig. 12c). Durability tests for WON-5/TiO_2_ revealed slight decay in its performance over the first 5 cycles probably due to deactivation of photocatalyst active sites by adsorbed degradation products (Fig. 12d).

**Scheme 1:**
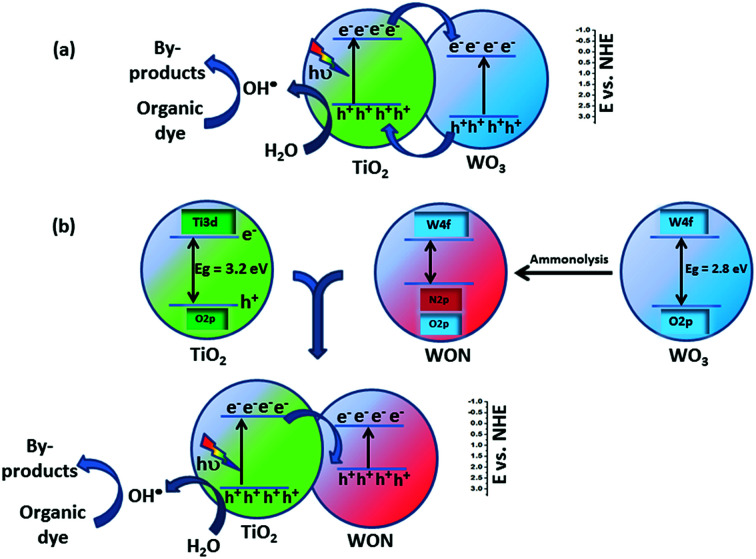
Charge transfer through (a) TiO_2_/WO_3_ heterojunction (b) TiO_2_/WON heterojunction.

The enhancement in photocatalytic activity of WON/TiO_2_ can be attributed to the charge transfer taking place at the heterojunction. In case of WO_3_/TiO_2_, both the valence band and conduction bands of WO_3_ exists at more positive potential compared to their counterparts of TiO_2_. The conduction band, CB of WO_3_ can act as a sink of electrons and capture the photo-generated CB electrons of TiO_2_ which lies at a potential of −0.33 eV *vs.* NHE,^64^ whereas, the valence band, VB of TiO_2_ acts as a sink of holes, so holes will transfer from VB of WO_3_ to the VB of TiO_2_ that lies at a potential of 2.95 eV *vs.* NHE and thus enhance electron–hole separation and hence enhance overall photocatalytic activity (Scheme 1a).^63,64^

After annealing of WO_3_ in ammonia, nitrogen N^3−^ replace O^2−^ in the lattice of the oxide and hence the VB of WON shifts to more negative potential than that of TiO_2_ as a result of partial substitution of oxygen with nitrogen accompanied with the contribution of N 2p in the valence band.^54,66–68^ Hence, the photo-induced holes in the VB of WON cannot transfer to the VB of TiO_2_ which lies at more negative potential. Interestingly, the enhanced photocatalytic activity of WON/TiO_2_ heterojunction compared to TiO_2_ can be ascribed to that the generated charge carriers (electrons) in the CB of TiO_2_ migrated to the VB WON and participated in the reduction reaction (Scheme 1b). Such mechanism was assumed by many authors to account for photoactivity of different heterojunctions.^69,70^ The redox potential of (O_2_/·O_2_^−^) is −0.33 eV *vs.* NHE which is more negative than CB energy of WON, hence the accumulated electrons on the CB of WON cannot reduce O_2_ to form ·O_2_^−^. On the other hand, the redox potential of (H_2_O/·OH) is 2.4 eV *vs.* NHE which is less positive than the VB of TiO_2_ (2.95 V *vs.* NHE) so ·OH radicals are the expected formed active species.^64,71,72^

## Conclusions

WON/TiO_2_ nanocomposite was prepared by coupling of electrospun and annealed TiO_2_ nanofibers with WON synthesized by ammonia annealing of WO_3_. The nanocomposite displayed lower charge transfer resistance and enhanced photocatalytic activity compared to TiO_2_ and WON counterparts. WON-5/TiO_2_ expressed the optimum composition with best performance towards simulated sunlight assisted-photodegradation of MO, MB, and phenol. The photoactivity of WON/TiO_2_ can be attributed to the enhanced separation of electron–hole pairs, high surface area and porosity, and decreased band gap energy.

## Conflicts of interest

There are no conflicts to declare.

## Supplementary Material

RA-008-C8RA06477F-s001
