# Supplementary material for: Enhanced photocatalytic performance of WON@porous TiO2 nanofibers towards sunlight-assisted degradation of organic contaminants
Source: RSC Adv. 2018 Sep 21;8(57):32747–55. doi: 10.1039/c8ra06477f (PMC9086300; doi:10.1039/c8ra06477f)
Supplement: RA-008-C8RA06477F-s001 [file RA-008-C8RA06477F-s001.pdf]

Electronic Supplementary Material (ESI) for RSC Advances.
This journal is © The Royal Society of Chemistry 2018

**Supporting information**

# Enhanced Photocatalytic Performance of WON@Porous $TiO_2$ Nanofibers towards Sunlight-Assisted Degradation of Organic Contaminants

Yahia H. Ahmad,[a] Assem M. Taha,[a] Mostafa H. Sliem,[b] Aboubakr M. Abdullah,[b] and Siham Y. Al-Qaradawi,[a*]

[a.] *Department of Chemistry and Earth Sciences, College of Arts and Sciences, Qatar University, Doha 2713, Qatar. E-mail: siham@qu.edu.qa*
[b.] *Center for Advanced Materials, Qatar University, Doha 2713, Qatar*

Fig. S1 presents the Raman spectra of $TiO_2$ NFs, WON, and WON/$TiO_2$. Anatase has six characteristic Raman active modes, $A_{1g}$, $2B_{1g}$, and $3E_g$. Well resolved Raman peak can be observed at 143 $cm^{-1}$ assigned to anatase vibrational mode $E_{1g}(1)$. Raman peak observed at 516 $cm^{-1}$ attributed to $A_{1g}$ and $B_{1g}(2)$. Other peaks at 196, 397, and 640 $cm^{-1}$ designated to $E_g(2)$, $B_{1g}(1)$, and $E_g(3)$, respectively. Raman spectrum of WON revealed peaks at 128, 185, 260, 321, 700, and two combined peaks at 800 and 809 $cm^{-1}$. Peaks at 128 and 185 are assigned to lattice vibrations, those at 260 and 321 $cm^{-1}$ to bending vibrations of O-W-O in the oxynitride, whereas the peaks at 795-805 $cm^{-1}$ arise from stretching vibrations of O-W-O. All peaks corresponds different modes of vibrations in the corresponding oxide but broadened and shifted to lower wavenumbers as a result of oxygen loss from $WO_3$ structure with formation of new W-N bonds therefore increases the bond length in the oxynitride compared to the oxide counterpart. Upon coupling of $TiO_2$ NFs with WON, the Raman modes of $TiO_2$ dominates with slight shift of peaks to higher wavenumber which confirms the interaction between $TiO_2$ and WON (Fig. S2). [59]

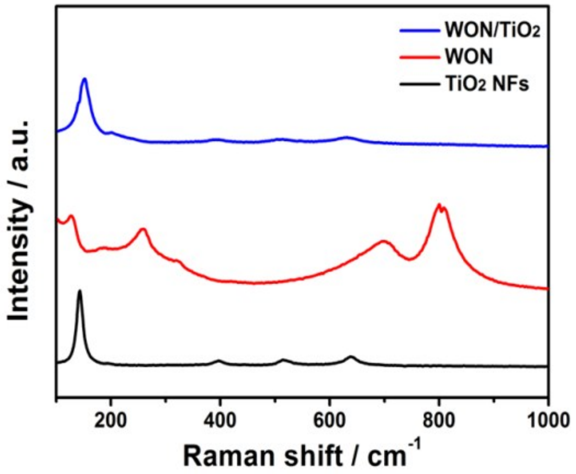


**Fig. S1.** Raman spectra of $TiO_2$ NFs, WON, and WON/$TiO_2$.

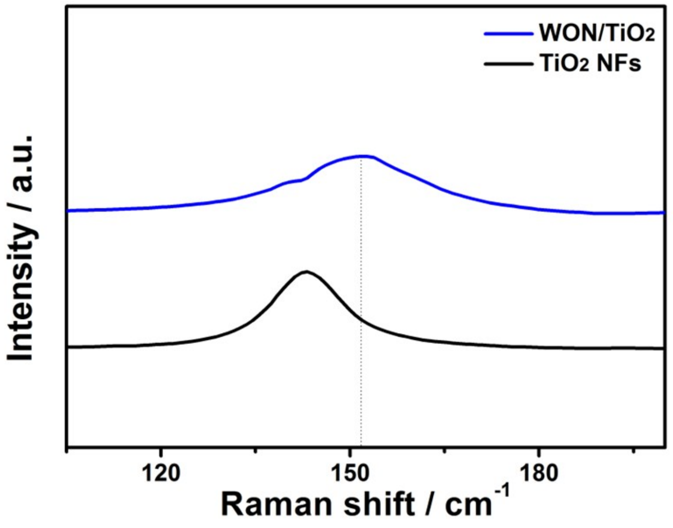


**Fig. S2**. Raman spectra of $TiO_2$ NFs compared to WON/$TiO_2$

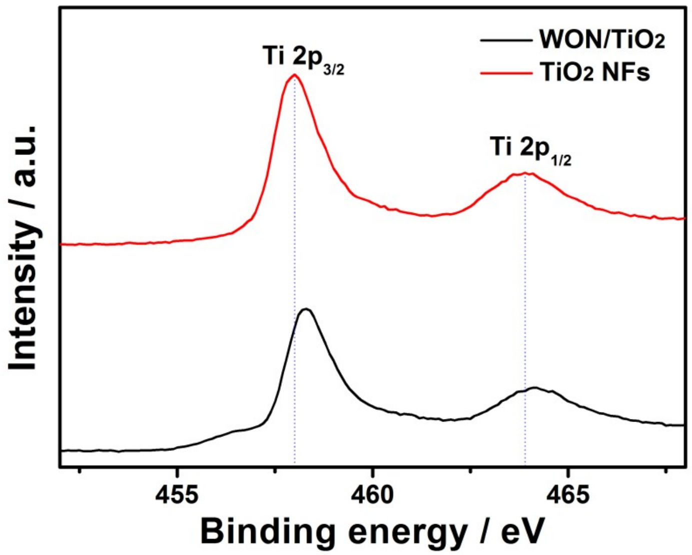


**Fig. S3**. (a) High resolution XPS spectra of Ti 2p in $TiO_2$ NFs and WON/$TiO_2$.

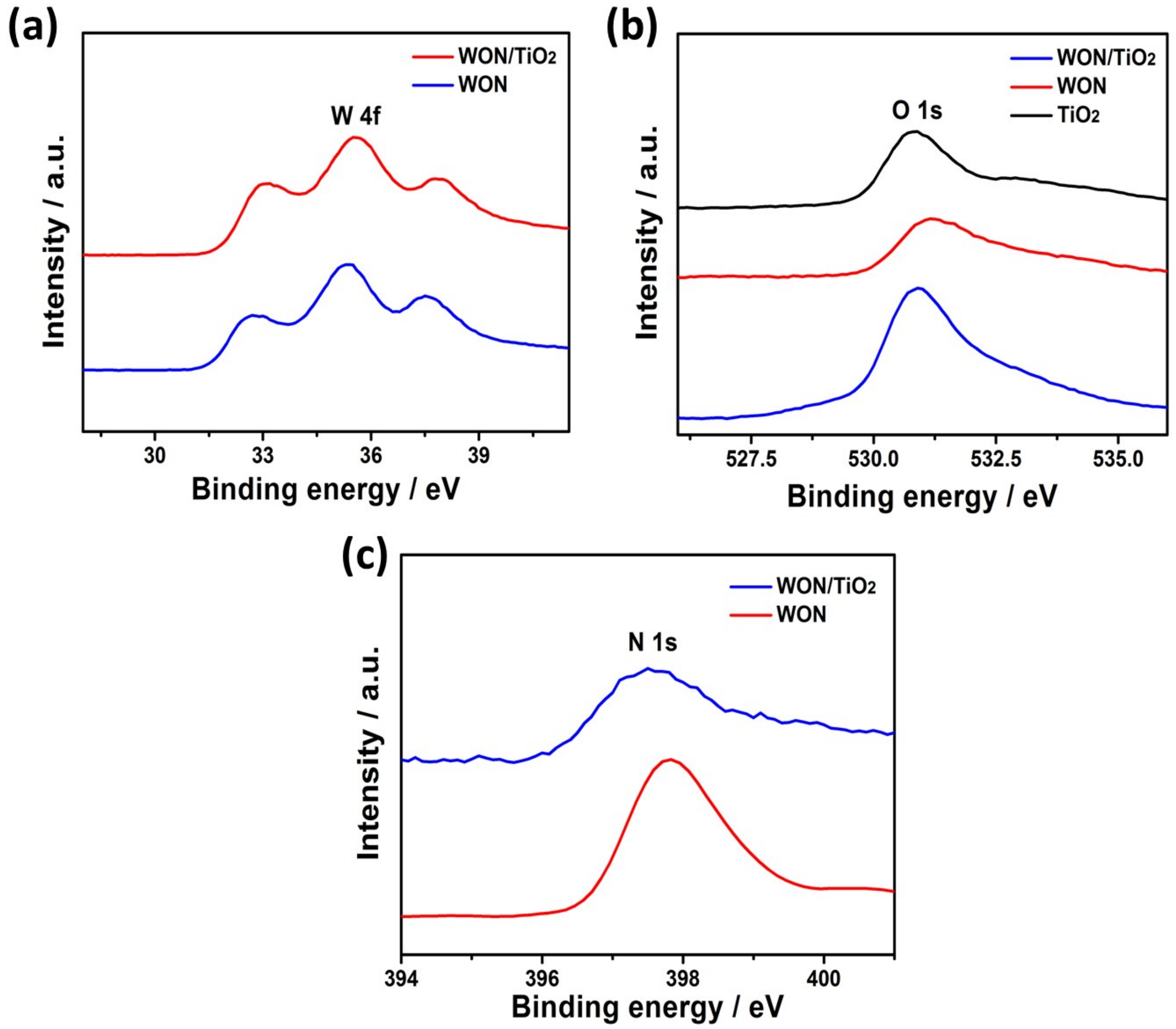


**Fig. S4**. (a) High resolution XPS spectra of W 4f, O 1s, and N 1s of different photocatalysts.
